# Population-based Surveillance for Bacterial Meningitis in China, September 2006–December 2009

**DOI:** 10.3201/eid2001.120375

**Published:** 2014-01

**Authors:** Yixing Li, Zundong Yin, Zhujun Shao, Manshi Li, Xiaofeng Liang, Hardeep S. Sandhu, Stephen C. Hadler, Junhong Li, Yinqi Sun, Jing Li, Wenjing Zou, Mei Lin, Shuyan Zuo, Leonard W. Mayer, Ryan T. Novak, Bingqing Zhu, Li Xu, Huiming Luo, the Acute Meningitis and Encephalitis Syndrome Study Group

**Keywords:** bacterial meningitis, meningitis, pneumococcal, Neisseria meningitidis, Streptococcus pneumoniae, Haemophilus influenzae type b, bacteria, China, population-based surveillance

## Abstract

During September 2006–December 2009, we conducted active population and sentinel laboratory–based surveillance for bacterial meningitis pathogens, including *Streptococcus pneumoniae, Neisseria meningitidis,* and *Haemophilus influenzae* type b, in 4 China prefectures. We identified 7,876 acute meningitis and encephalitis syndrome cases, including 6,388 among prefecture residents. A total of 833 resident cases from sentinel hospitals met the World Health Organization case definition for probable bacterial meningitis; 339 of these cases were among children <5 years of age. Laboratory testing confirmed bacterial meningitis in 74 of 3,391 tested cases. The estimated annual incidence (per 100,000 population) of probable bacterial meningitis ranged from 1.84 to 2.93 for the entire population and from 6.95 to 22.30 for children <5 years old. Active surveillance with laboratory confirmation has provided a population-based estimate of the number of probable bacterial meningitis cases in China, but more complete laboratory testing is needed to better define the epidemiology of the disease in this country.

## Abstract 2

Greater use of appropriate specimen collection and confirmatory laboratory testing is needed.

Bacterial meningitis continues to be a major cause of illness and death in neonates and children throughout the world (*1*). The leading vaccine-preventable causes of this disease are *Streptococcus pneumoniae*, *Neisseria meningitidis*, and *Haemophilus influenzae* type b (Hib). The increasing availability of vaccines has made public health initiatives to control bacterial meningitis disease feasible; however, the incidence of this disease and associated deaths in resource-limited countries continue to exceed those for developed countries (*2*). Accurate pathogen-specific estimates of the number of bacterial meningitis cases are needed to monitor and refine vaccination programs, but such estimates are challenging to obtain in many countries because of limited laboratory-based surveillance capacity.

Thirty-nine infectious diseases are currently routinely reportable in China. However, among the common bacterial meningitis pathogens, only epidemic meningitis caused by *N. meningitidis* is reportable (*3*,*4*). Most reported cases of epidemic meningitis represent clinical diagnoses that lack laboratory confirmation because of the low culture rate for *N. meningitidis.*

In population-based studies, the incidence of acute bacterial meningitis in China ranges from 12.4 to 19.2 cases/100,000 children <5 years of age (*5*,*6*). Available studies suggest that the main causal pathogens of bacterial meningitis in China are *N. meningitidis*, Hib, and *S. pneumoniae* (*7*–*9*). In population-based studies in Hefei and Nanning, China, the incidence of meningitis caused by Hib was 10.66 and 0.98 cases/100, 000 children <5 years of age, respectively (*6*,*10*), and the incidence of *S. pneumoniae* meningitis was 1.5 and 1.3 cases/100,000 children <5 years of age, respectively (*6*,*11*).

In 2006, the Acute Meningitis and Encephalitis Syndrome Project was initiated in China (*12*,*13*) to determine the incidence and epidemiology of vaccine-preventable causes of meningitis and encephalitis and to improve the laboratory capacity for diagnosis of these diseases. Using data from the project’s active surveillance system, we report the etiology, epidemiology, and estimated incidence of bacterial meningitis in 4 Chinese prefectures during September 2006–December 2009. 

## Materials and Methods

### Disease Surveillance

**Figure 1:**
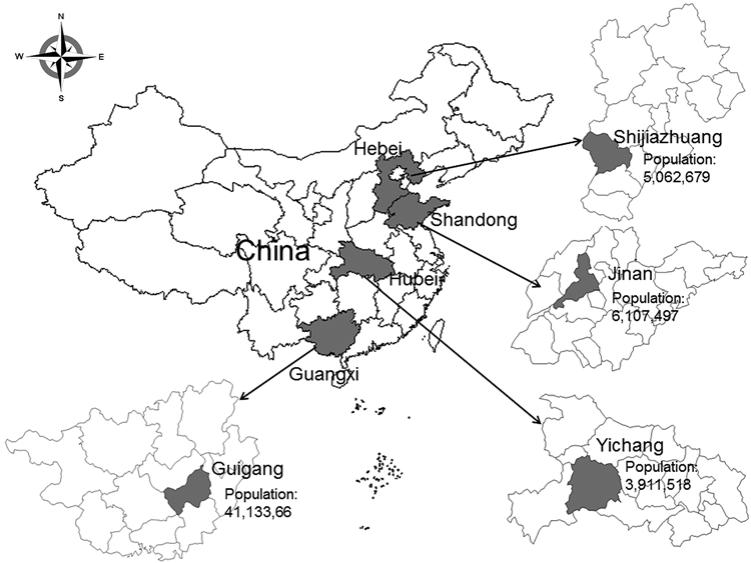
Location and population of 4 prefectures in China in which active population and sentinel laboratory–based surveillance for bacterial meningitis pathogens was conducted through the Acute Meningitis and Encephalitis Syndrome Surveillance System during September 2006–December 2009. The province in which each prefecture is located is indicated on the main map.

The Acute Meningitis and Encephalitis Syndrome Project was launched in September 2006 in Jinan Prefecture, the capital of Shandong Province, and Yichang Prefecture, which is in western Hubei Province (Figure 1). In April 2007, the project areas were expanded to Shijiazhuang Prefecture, the capital of Hebei Province, and Guigang Prefecture, which is in southeast Guangxi Province. Detailed methods are reported elsewhere (*12*). In brief, 6 hospitals were selected as sentinel hospitals in each prefecture, including the largest general infectious disease and children’s hospitals. Efforts were made to collect diagnostic specimens (serum and CSF) for laboratory testing from all clinical cases of meningitis and encephalitis syndrome. All other hospitals in each prefecture were designated as nonsentinel hospitals, at which epidemiologic data, but not specimens, were collected on reported acute meningitis and encephalitis syndrome (AMES) cases (*13*).

An AMES case-patient was defined as a person of any age who experienced acute onset of fever with change in mental status and/or meningeal signs (e.g., neck stiffness, headache). AMES case-patients were considered to have probable bacterial meningitis (PBM) if laboratory test results demonstrated at least 1 of the following: a turbid appearance of CSF; leukocytosis (>100 cells/mm^3^; reference 0–8 cells/mm^3^); or leukocytosis (10–100 cells/mm^3^) and elevated level of protein (>100 mg/dL; reference 20–40 mg/dL) or decreased glucose (<40 mg/dL; reference 50–80 mg/dL). These signs are consistent with the World Health Organization (WHO) PBM case definition (*14*). Case-patients with any blood or CSF laboratory test results positive for specific bacterial pathogens were defined as confirmed bacterial meningitis case-patients.

Clinic doctors in sentinel and nonsentinel hospitals reported cases of illness meeting the AMES case definition to the county Center for Disease Control and Prevention (CDC) through passive surveillance. Public health doctors performed case investigations within 48 hours after receiving case reports from sentinel hospitals, and they collected case information, including date of birth, date of symptom onset, and initial clinical diagnosis, for cases in nonsentinel hospitals.

Active surveillance was performed every 10 days in both sentinel and nonsentinel hospitals. Public health doctors reviewed admission and discharge records in the pediatric, neurology, and infectious disease inpatient and outpatient departments and queried clinic doctors to identify and report any missed AMES cases. Computer files in sentinel hospitals were also reviewed to identify all cases with 12 specific initial diagnostic codes: Japanese encephalitis (JE), viral encephalitis, viral meningitis, viral meningoencephalitis, other encephalitis, meningococcal meningitis, purulent meningitis, cerebrospinal meningitis, TB meningitis, TB meningoencephalitis, other meningitis, and other diagnosis. The key indicators for surveillance quality were that blood and CSF specimens were obtained from >90% and 70%, respectively, of AMES case-patients.

### Specimen Collection and Laboratory Testing

For this study, serum and cerebrospinal fluid (CSF) specimens were collected from patients at the sentinel hospitals as part of routine diagnostic testing. Testing performed at reference laboratories was done on de-identified specimens. CSF specimens were collected in hospitals with the patient’s or parent’s signed informed consent. The ethical review committee of the Chinese CDC determined that additional informed consent was not required because this study was for surveillance and used standard clinical specimens.

Physicians in sentinel hospitals collected blood (>3 mL) and/or CSF specimens from the AMES case-patients. For CSF specimens, routine examination, including the leukocyte count and glucose and protein levels, was conducted by sentinel hospital laboratories according to the usual physicians’ requests; latex agglutination, bacterial culture of CSF, and blood cultures were performed at the physician’s discretion. Two kinds of latex agglutination reagents (bioMérieux, Marcy-l’Etoile, France, and Bio-Rad, Marnes-la-Coquette, France) were used according to the manufacturers’ guidelines. All specimens (serum or CSF) were stored at or below −20°C and transported to prefecture CDC laboratories and, subsequently, to provincial CDC laboratories. Prefecture CDC laboratories performed JE virus IgM ELISA testing on all CSF and serum specimens (*12*).

Bacterial isolates from hospital laboratories were transported to the provincial CDC laboratories for confirmatory testing and testing for antimicrobial drug susceptibility. These bacteriology laboratories tested CSF specimens for *N. meningitidis*, *H. influenzae*, and *S. pneumoniae* by using real-time PCR. Staff in all laboratories had received training from US Centers for Disease Control and Prevention and Chinese CDC bacteriologists. The primers have been described (*15*,*16*). Shandong and Hubei provincial CDC laboratories tested all CSF specimens that were negative for JE; other virus testing for Jinan and Yichang Prefectures was done at the Shandong and Hubei laboratories, respectively. Hebei and Guangxi provincial CDC laboratories tested all PBM case specimens according to the WHO definition noted above (*14*)*.* A subset of specimens and isolates was forwarded to the Chinese CDC bacterial laboratory (Beijing) to confirm PCR results and to analyze the strains by using multilocus sequence typing.

### Data Collection and Analysis

Demographic, clinical, and laboratory data from the sentinel hospitals were entered in an EpiData database (http://epidata.dk/). Data from nonsentinel hospitals were compiled by using standardized Excel spreadsheet software (Microsoft, Redmond, WA, USA).

To estimate the PBM incidence in the 4 prefectures, we first calculated the proportions of PBM cases among resident AMES case-patients who had CSF specimens collected in sentinel hospitals, stratified by age and initial diagnosis. We then multiplied these proportions by the number of resident cases (tested and untested in sentinel and nonsentinel hospitals), again stratified by age and initial diagnosis, and divided by the prefecture population under surveillance. The χ^2^ test was used to determine significance of differences in rates and percentages.

## Results

### Overall Surveillance by Province

A total of 7,876 AMES cases were reported, 4,712 from sentinel hospitals and 3,164 from nonsentinel hospitals (Table 1). The percentage of residents among AMES case-patients varied by location of the prefectures and between sentinel and nonsentinel hospitals; >50% of cases in sentinel hospitals in 2 prefectures were in persons from outside the prefecture (*12*). Overall, the incidences of AMES cases for prefecture residents were similar; in general, there were ≈10 cases/100,000 residents (range 9.1–13.5 cases/100,000). However, the rate was higher among children <5 years of age, among whom AMES incidence among prefecture residents ranged from 30.8 to 96.9 cases/100,000 children (Table 1). 

**Table 1:** Surveillance for AMES cases in 4 China prefectures, September 2006–December 2009*

Variable	Province/prefecture				Total
	Shandong/Jinan	Hubei/Yichang	Hebei/Shijiazhuang†	Guangxi/Guigang	
Total population (no. <5 y of age)	6,107,497 (353,672)	3,911,518 (236,036)	5,062,679 (314,180)	4,113,366 (261,302)	
Project duration, mo	40	40	32	32	
Total no. cases	2,647	1,631	2,093	1,505	7,876
Cases in sentinel hospitals, no. (%)	1,200	1,055	959	1,498	4,712
Residents	607 (50.6)	979 (92.8)	439 (45.8)	1,474 (98.4)	3,499 (74.3)
Nonresidents	593 (49.4)	76 (7.2)	520 (54.2)	24 (1.6)	1,213 (25.7)
Residents <5 y of age	170 (44.2)	161 (92.5)	149 (37.2)	671 (98.4)	1,151 (70.1)
Nonresidents <5 y of age	215 (55.8)	13 (7.5)	252 (62.8)	11 (1.6)	491 (29.9)
Cases in nonsentinel hospitals, no. (%)	1,447	576	1,134	7	3,164
Residents	1,241 (85.8)	574 (99.7)	1,067 (94.1)	7 (100.0)	2,889 (91.3)
Nonresidents	206 (14.2)	2 (0.3)	67 (5.9)	0 (0)	275 (8.7)
Residents <5 y of age	193 (87.7)	105 (100.0)	360 (96.0)	4 (100.0)	662 (94.0)
Nonresidents <5 y of age	27 (12.3)	0 (0)	15 (4.0)	0 (0)	42 (6.0)
Cases among prefecture residents per year/100,000 persons					
Residents	9.08	11.91	11.16	13.50	
Residents <5 y of age	30.79	33.81	60.75	96.87	

*AMES, acute meningitis and encephalitis syndrome. †Only 13 counties in Shijiazhuang Prefecture were included in the project.

### Demographic Features of the AMES Cases and Bacterial Testing

Among the AMES case-patients identified in sentinel hospitals, 42.2%–81.7% were children <15 years of age, but age distribution differed between the 4 prefectures (Table 2). CSF specimens were obtained from 60.0%–83.9% of case-patients in the 4 prefectures. Bacterial testing rates for cases in sentinel hospitals varied by testing method and prefecture; CSF culture, CSF PCR, and blood culture were the most frequently used methods. Use of CSF culture ranged from 20.8% in Jinan Prefecture to 53.7% in Yichang Prefecture, and use of CSF real-time PCR ranged from 13.7% in Shijiazhuang Prefecture to 60.1% in Jinan. Use of any testing method for bacterial pathogens ranged from 58.7% to 79.1% in the 4 prefectures (Table 2).

**Table 2:** Demographic features and bacterial laboratory testing for AMES case-patients in sentinel hospitals in 4 China prefectures, September 2006–December 2009*

Age, sex, and laboratory testing for case-patients	No. (%) patients by province/prefecture				Total no (%), n = 4,712
	Shandong/Jinan, n = 1,200	Hubei/Yichang n = 1,055	Hebei/Shijiazhuang, n = 959	Guangxi/Guigang, n = 1,498	
Case-patient age, y					
<2	152 (12.7)	85 (8.1)	232 (24.2)	387 (25.8)	856 (18.2)
2–4	233 (19.4)	89 (8.4)	169 (17.6)	295 (19.7)	786 (16.7)
5–14	393 (32.8)	271 (25.7)	383 (39.9)	380 (25.4)	1,427 (30.3)
15–29	170 (14.2)	189 (17.9)	86 (9.0)	110 (7.3)	555 (11.8)
30–44	135 (11.3)	197 (18.7)	33 (3.4)	88 (5.9)	453 (9.6)
>45	117 (9.8)	224 (21.2)	56 (5.8)	238 (15.9)	635 (13.5)
Case-patient sex					
M	741 (61.8)	642 (60.9)	586 (61.1)	936 (62.5)	2,905 (61.7)
F	459 (38.3)	413 (39.1)	373 (38.9)	562 (37.5)	1,807 (38.3)
Case-patient specimen testing					
CSF examination	1,007 (83.9)	751 (71.2)	575 (60.0)	1,131 (75.5)	3,464 (73.5)
Blood, first culture	26 (2.2)	507 (48.1)	311 (32.4)	662 (44.2)	1,506 (32.0)
CSF culture	250 (20.8)	567 (53.7)	345 (36.0)	754 (50.3)	1,916 (40.7)
CSF latex agglutination	80 (6.7)	282 (26.7)	95 (9.9)	286 (19.1)	743 (15.8)
CSF real-time PCR	721 (60.1)	540 (51.2)	131 (13.7)	407 (27.2)	1,799 (38.2)
Any testing method	851 (70.9)	834 (79.1)	563 (58.7)	1,143 (76.3)	3,391 (72.0)
No. case-patients positive for Japanese encephalitis virus	136	29	20	111	296

*AMES, acute meningitis and encephalitis syndrome; CSF, cerebrospinal fluid.

### Frequency of PBM and Results of Bacterial Testing

Overall in sentinel hospitals, 833/3,464 (24.0%; percentages ranged from 15.8% for Yichang Prefecture to 31.0% for Shijiazhuang Prefecture) of AMES cases with CSF specimens met the WHO definition of PBM (*14*) (Table 3); 339 (40.7%) occurred in children <5 years of age. Among the AMES case-patients, children <5 years of age were more likely than older patients to have PBM (29.1% vs. 21.5%).

**Table 3:** Demographic information for AMES case-patients meeting the World Health Organization definition for probable bacterial meningitis, China, September 2006–December 2009*

Demographic variable	% PMB case-patients (no. with CSF specimen/no. total), by province/prefecture				% total PBM case-patients (no. with CSF specimen/no. total), n = 3,464
	Shandong/Jinan, n = 1,007	Hubei/Yichang, n = 751	Hebei/Shijiazhuang, n = 575	Guangxi/Guigang, n = 1,131	
Total PBM patients	28.6 (288/1,007)	15.8 (119/751)	31.0 (178/575)	21.9 (248/1,131)	24.0 (833/3,464)
Case-patient age, y					
<2	28.9 (39/135)	26.8 (11/41)	47.0 (95/202)	22.0 (54/246)	31.9 (199/624)
2–4	29.5 (62/210)	16.7 (8/48)	25.0 (23/92)	24.7 (47/190)	25.9 (140/540)
5–14	30.6 (102/333)	16.1 (25/155)	20.9 (37/177)	20.8 (57/274)	23.5 (221/939)
15–29	23.3 (31/133)	14.1 (22/156)	28.1 (16/57)	20.4 (21/103)	20.0 (90/449)
30–44	28.4 (29/102)	17.1 (28/164)	19.0 (4/21)	24.7 (21/85)	22.0 (82/372)
>45	26.6 (25/94)	13.4 (25/187)	11.5 (3/26)	20.6 (48/233)	18.7 (101/540)
Case-patient sex					
M	30.3 (191/630)	17.1 (79/463)	30.7 (107/349)	20.9 (147/705)	24.4 (524/2,147)
F	25.7 (97/377)	13.9 (40/288)	31.4 (71/226)	23.7 (101/426)	23.5 (309/1,317)
Case-patient place of residence					
Residents	31.9 (166/521)	15.4 (106/687)	25.7 (45/175)	21.6 (241/1,116)	22.3 (558/2,499)
Non-residents	25.1 (122/486)	20.3 (13/64)	33.3 (133/400)	46.7 (7/15)	28.5 (275/965)
Yearly PBM incidence/100,000 population					
All residents					
Crude rate	0.82	0.81	0.33	2.20	
Adjusted rate*	2.61	1.84	2.26	2.93	
Residents <5 y old					
Crude rate	4.16	2.16	3.22	14.21	
Adjusted rate†	8.41	6.95	14.47	22.30	

*AMES, acute meningitis and encephalitis syndrome; PBM, probable bacterial meningitis. †Adjusted rate was calculated based on PBM positivity among resident case-patients by age and initial clinical diagnosis applied to all cases without cerebrospinal fluid specimen and profile. Two assumptions were made when adjusting the rate: (1) the subset tested is a representative specimen of AMES cases, and (2) there was a homogenous rate of infection within a given prefecture, age group, and initial clinical diagnosis stratum.

The proportion of AMES cases attributed to PBM in each age group also varied by prefecture. In Yichang, only 19 (16.0%) of 119 PBM cases were in children <5 years of age. However, in other prefectures, a large proportion of cases occurred in this age group. In Jinan, 101 (35.1%) of 288 PBM cases were in children <5 years of age, and in Shijiazhuang, 118 (66.3%) of 178 PBM cases were in children <5 years of age.

**Figure 2:**
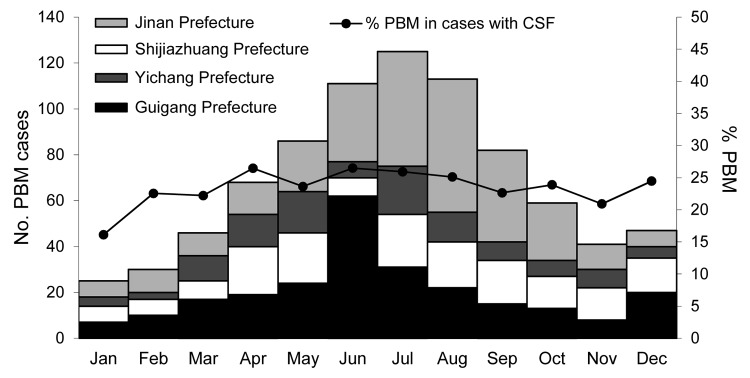
Number and percent of probable bacterial meningitis (PBM) cases by month among acute meningitis and encephalitis syndrome case-patients in 4 China prefectures, September 2006–December 2009.

PBM cases were detected each month during the study period (Figure 2), but there was a slight peak in cases during June–August. In the 2 prefectures with the most marked seasonality (Jinan and Guigang), the peaks occurred during July–September and June–July, respectively, consistent with peak periods for JE cases (*12*).

The crude incidence rates of PBM ranged from 0.33 cases/100,000 persons in Shijiazhuang to 2.20 cases/100,000 persons in Guigang. After adjustment based on PBM case positivity rates by age and initial diagnosis, the estimated PBM incidence rate in the 4 prefectures was ≈2 cases/100,000 persons (range 1.84 cases/100,000 persons for Yichang to 2.93 cases/100,000 persons for Guigang). Among children <5 years of age, the estimated PBM incidence in the prefectures varied >3-fold, from 6.95 to 22.30 cases/100,000 children for Yichang and Guigang, respectively (Table 3).

Among 3,391 case-patients to have clinical specimens tested by any bacterial detection method, 74 (2.2%) were laboratory confirmed to have bacterial meningitis: 26 cases caused by *N. meningitidis*, 9 cases caused by Hib, and 39 cases caused by *S. pneumoniae*. Among confirmed cases, 18 (24.3%) were confirmed by CSF culture and 62 (83.8%) by real-time PCR. Confirmation rates by real-time PCR were higher than those by any other method (3.4% vs. 0.2%–1.3%; p<0.01) (Table 4).

**Table 4:** Bacterial testing results for specimens from AMES case-patients, China, September 2006–December 2009*

Test	No. cases positive/no. tested	% Positive	No. cases positive, by pathogen		
			*Neisseria meningitidis*†	*Haemophilus influenzae* type b	*Streptococcus pneumoniae*
Blood culture		0.2	3	0	0
CSF culture	3/1,506	0.9	1	5	12
Latex agglutination	18/1,916	1.3	6	1	3
Real-time PCR	10/743	3.4	25	7	30
Any method	62/1,799	2.2	26	9	39

*AMES, acute meningitis and encephalitis syndrome; CSF, cerebrospinal fluid. †One case was serogroup A, 3 were serogroup B, and 18 were serogroup C; serogroup could not be determined for 4 cases

AMES cases with an initial clinical diagnosis of meningococcal meningitis or purulent meningitis were more likely than cases with other initial diagnoses to meet the WHO case definition for PBM (41.7%) and to have a higher rate of laboratory confirmation for any 1 of the 3 bacterial pathogens (11.9%) (Table 5). Of the 74 laboratory-confirmed cases, 34 (45.9%) had an initial clinical diagnosis of meningococcal meningitis or purulent meningitis, and the remaining 40 (54.1%) cases had an initial diagnosis of some other clinical syndrome or etiology (Table 5). The proportion of laboratory-confirmed bacterial cases among the PBM cases was higher than among the other tested AMES cases (5.5% vs. 0.8%, respectively; p<0.01).

**Table 5:** Initial clinical diagnoses of AMES and probable bacterial meningitis cases in sentinel hospitals in 4 China prefectures, September 2006–December 2009*

Initial diagnosis	No. (%) AMES cases	No. AMES cases with CSF specimen/no. (%) meeting WHO definition of PBM	No. AMES specimens tested/no. (%) confirmed positive for any bacteria
Meningococcal meningitis	19 (0.4)	13/6 (46.2)	12/5 (41.7)
Purulent meningitis	280 (5.9)	255/156 (61.2)	244/29 (11.9)
TB meningitis	149 (3.2)	132/55 (41.7)	114/1 (0.9)
TB meningoencephalitis	48 (1.0)	33/17 (51.5)	31/0
Japanese encephalitis	142 (3.0)	102/29 (28.4)	110/0
Viral encephalitis	1,631 (34.6)	1,173/221 (18.8)	1,146/12 (1.0)
Viral meningitis	231 (4.9)	179/38 (21.2)	170/3 (1.8)
Viral meningoencephalitis	181 (3.8)	137/31 (22.6)	135/2 (1.5)
Other encephalitis	1,201 (25.5)	842/155 (18.4)	814/12 (1.5)
Other meningitis	70 (1.5)	57/14 (24.6)	55/0
Cerebrospinal meningitis	10 (0.2)	8/2 (25.0)	8/1 (12.5)
Other diagnosis†	731 (15.5)	526/109 (20.7)	539/9 (1.70)
Data missing	19 (0.4)	7/0	13/0
Total	4,712 (100)	3,464/833 (24.0)	3,391/74 (2.2)

*AMES, acute meningitis and encephalitis syndrome; CSF, cerebrospinal fluid; WHO, World Health Organization; PBM, probable bacterial meningitis; TB, tuberculosis. †Other diagnosis were cases that met the AMES case definition (e.g., suspected CNS infection and high fever and convulsion with unknown reason) but that were not included among the 11 specific initial diagnosis listed above.

The epidemiologic features of laboratory confirmed bacterial meningitis varied by pathogen. Confirmed *N. meningitidis* cases were predominantly serogroup C (18 [69%] cases) (Table 4). Half of the confirmed *N. meningitidis* case-patients were <15 years of age. Confirmed *S. pneumoniae* cases were present in each age group, but predominately among children <2 years of age (12 [30.8%] cases) and in persons >45 years of age (8 [20.5%] cases). The confirmed Hib cases were all in children <2 years of age. Among children in this age group, *S. pneumoniae* was the predominant pathogen (12 cases) followed by Hib (9 cases) and *N. meningitidis* (3 cases).

The 74 confirmed bacterial meningitis cases occurred across all months; however, 22 (85%) *N. meningitidis* cases occurred in the first half of the year. Hib and *S. pneumoniae* cases occurred in all seasons.

## Discussion

During September 2006–December 2009, we identified >7,000 AMES cases in 4 China prefectures. The overall incidence was ≈10 cases/100,000 residents, and the estimated PBM incidence was ≈2 cases/100,000 persons for the entire population and 7–22 cases/100,000 children for residents <5 years of age. Using a variety of laboratory testing methods, we confirmed 74 bacterial meningitis cases among the AMES cases; real-time PCR had the highest positivity rate among the methods used for pathogen testing.

*N. meningitidis*, *S. pneumoniae*, Hib, and JE virus are serious but vaccine-preventable causes of meningitis and encephalitis in China. Surveillance for bacterial meningitis and viral encephalitis has previously been conducted separately, and only JE and *N. meningitidis* (epidemic meningitis) are reportable diseases (*12*). In our study, CSF specimens were obtained from 73.5% of AMES case-patients in sentinel hospitals in the 4 prefectures. This percentage exceeds the surveillance indicator for CSF specimen collection (i.e., 70% among all AMES cases) and shows the feasibility of using active and passive surveillance with sentinel laboratory testing, as used in our study population, to detect bacterial meningitis.

PBM cases were reported during all months in each of the 4 prefectures, but there was a slight peak during June–August in Jinan and Guigang Prefectures, locations that had most of the confirmed cases of JE during the JE epidemic season (*12*). The percentage of PBM cases among the AMES case-patients with CSF specimens varied substantially by age group among the 4 prefectures. These differences may be due to several factors, including variations in ecologies, population characteristics, and the use of vaccines. In 1986, Shandong Province integrated meningococcal polysaccharide A vaccine into its provincial Expanded Program on Immunization and since then has achieved systematic vaccination of children. The Hib conjugate vaccine was available for children in the 4 prefectures studied, but its use was generally limited to urban areas, and parents had to pay for the vaccine. *S. pneumoniae* vaccine was introduced in China at the end of 2008, but its use is limited because the vaccine price is extremely high (*17*).

We estimated that, during September 2006–December 2009, the annual incidence of PBM among the population of the 4 prefectures studied was ≈2 cases/100,000 persons. For children <5 years of age, the estimated incidence (6.95–22.3 cases/100,000 children) was lower than that for Poland and Bulgaria before Hib vaccine was introduced (29 and 35 cases/100,000 children, respectively) (*18*,*19*). Our estimated incidence among children <5 years of age was also lower than that for Mongolia (68 cases/100,000 children 2–5 years of age) and Korea (91 cases/100,000 children <5 years of age) (*20*,*21*), but the incidence in our study was similar to that found in other studies in China (*5*,*6*) and other Asian countries, such as Thailand (27 cases/100,000 children <5 years of age) (*22*). The lower incidence among children <5 year of age in our study may be due to regional factors, wide use of *N. meningitidis* vaccine, and moderate use of the Hib vaccine in the 4 prefectures.

*N. meningitidis*, Hib, and *S. pneumoniae* are the most common causes of acute bacterial meningitis; most studies indicate that these pathogens are responsible for >75% of all cases of bacterial meningitis overall and for 90% of cases in children (*23*). In our study, the relative proportion of cases caused by *N. meningitidis* (35.1%) was lower than that in a previous study in China (48.4% in Beijing) (*7*), and the proportion of cases caused by *S. pneumoniae* (52.7%) was relatively higher. For children <2 years of age, the predominant pathogens causing bacterial meningitis were *S. pneumoniae* and Hib. The lower proportion of *N. meningitidis*–related meningitis cases among young children may be the result of the wide use of the meningococcal polysaccharide vaccine in this age group.

By comparing the initial clinical diagnoses in our study with the bacterial meningitis laboratory results, we found that some clinical diagnoses (e.g., meningococcal meningitis and purulent meningitis) were most likely to represent cases with a confirmed bacterial cause. However, more than half of laboratory-confirmed bacterial meningitis cases had other initial clinical diagnoses. These findings point to a need to conduct sensitive laboratory testing for all suspected AMES case-patients, particularly for those meeting the WHO PBM case definition (*14*). Moreover, 12.2% of the laboratory-confirmed bacterial meningitis cases initially had another diagnosis, indicating the challenges posed trying to encompass all clinical diagnoses relevant for syndromic surveillance of bacterial meningitis.

Overall, the percentage of AMES cases confirmed as caused by *N. meningitidis*, *S. pneumoniae*, or Hib (2.2% of all tested cases and 9.0% of PBM cases) was substantially lower than the percentage in other studies (25% in Bangladesh for bacterial meningitis, 44.6% in Yemen for the acute meningitis) (*24*,*25*). The factors leading to low positive rates for bacterial meningitis were complex. One key factor is the indiscriminate use of antimicrobial drugs in clinical practice and by patients in China (*11*): 62% of all AMES case-patients and 66% of PBM case-patients in our study had used antimicrobial drugs before clinical specimens were collected. In addition, some case-patients were referred from smaller hospitals where conditions for specimen collection, storage, and transport may have also affected testing results. Other contributing factors were the enrollment of all clinically diagnosed meningitis and encephalitis case-patients, which likely included a wider range of viral pathogens, particularly JE, among cases that met the WHO PBM case definition, as well as some illnesses that were ultimately determined to not be of infectious etiology.

Among the laboratory methods we used for bacterial testing, real-time PCR had a substantially higher detection rate than other assays. This finding suggests the value of using real-time PCR to confirm suspected bacterial meningitis cases. Increased use of PCR will require making such tests more widely available in hospitals and public health laboratories in China and other countries attempting to evaluate bacterial causes of meningitis.

This study has several limitations. First, not all CSF specimens were tested by commonly used diagnostic methods (blood and CSF culture) in hospitals or by real-time PCR in the 4 provincial CDC laboratories. Testing in hospital clinical laboratories in China is done at the discretion of treating physicians and is often not done because of the limited ability of patients to pay for testing. In 2 provinces, only CSF specimens from patients with PBM were tested by PCR. Hence, our study likely missed many bacterial meningitis cases, particularly among case-patients whose illnesses did not meet the PBM case definition. Second, because of limited laboratory testing in nonsentinel hospitals, we relied on adjustment by clinical diagnosis to calculate estimated rates of PBM; inconsistent clinical diagnosis of nontested cases may have caused inaccuracies in these estimates. Last, because laboratory testing had low positivity rates for confirmed bacterial meningitis, we did not find enough confirmed cases to derive accurate incidence rates for each pathogen, thus limiting the generalizability of these data.

Despite the limitations, this project has provided useful insights into the incidence and epidemiology of bacterial meningitis in China. It is clear that active surveillance linked to laboratory confirmation is critical to estimate the number of bacterial meningitis cases. On the basis of our findings, we suggest that bacterial meningitis surveillance in China should be enhanced in several ways. First, wider use of appropriate specimen collection and handling is needed, along with laboratory testing to confirm the causative pathogens, especially for cases in young children. In the study setting, this enhanced surveillance should include ensuring appropriate specimen collection in all cases and not charging hospital patients for testing. These enhancements will require better training of physicians and hospital staff and more rigorous quality control regarding specimen collection. Second, real-time PCR is the most sensitive bacterial testing method and should be used in the sentinel sites. These efforts will help to improve active surveillance so that disease incidence and pathogen-specific etiologies for bacterial meningitis can be more accurately determined by age group and used to develop recommendations for the use of meningococcal conjugate vaccines and to determine the need for routine Hib and *S. pneumoniae* vaccinations for young children in China.
